# Digital versus Paper Reading: A Systematic Literature Review on Contemporary Gaps According to Gender, Socioeconomic Status, and Rurality

**DOI:** 10.3390/ejihpe13100142

**Published:** 2023-09-22

**Authors:** Igor Peras, Eva Klemenčič Mirazchiyski, Barbara Japelj Pavešić, Žiga Mekiš Recek

**Affiliations:** 1 Educational Research Institute, Gerbičeva ulica 62, 1000 Ljubljana, Slovenia; eva.klemencic@pei.si (E.K.M.); barbara.japelj@pei.si (B.J.P.); 2 Department of Psychology, Faculty of Arts, University of Ljubljana, Aškerčeva 2, 1000 Ljubljana, Slovenia

**Keywords:** digital reading, e-reading, paper reading, gender differences, socioeconomic status (SES), reading comprehension, literature review

## Abstract

This paper presents a comprehensive review of the literature on electronic reading (e-reading) versus paper reading. The main objective was to assess the current state of research comparing digital and paper reading outcomes among students aged 6–18 years old, as well as assessing the impact of various factors (gender, socioeconomic status, and school location) in explaining the differences between the two modes. Inclusion criteria included the following: participants (6–18 years), research focus (comparing digital reading and paper reading), study type (quantitative or mixed methods), publication (peer reviewed between 2015 and 2022), and language (English). A systematic search in four databases (WOS, Scopus, ERIC, and JSTOR) in August 2022 was conducted by three reviewers. The search revealed 23 studies matching the inclusion criteria. The findings from the reviewed studies are diverse, with some reporting no significant differences in reading comprehension between the two modes, while others suggest screen inferiority, thereby favoring paper reading. Individual-level predictors, such as prior comprehension skills and reading habits, play a crucial role in determining reading performance across modes. Family-level factors, such as the number of books at home, and school-level factors, like the usage of ICT resources, influence both paper and digital reading comprehension. Moreover, gender differences in attitudes and performance towards different reading modes are apparent. SES is positively associated with reading achievement in both modes, with a larger effect shown for paper reading. Overall, the comparison between electronic and paper reading modes reveals a complex interplay of individual and contextual factors influencing reading comprehension and attitudes.

## 1. Introduction

Technology has become a widespread and unavoidable factor in contemporary daily life. Devices such as desktop computers, laptops, tablets, and mobile phones have become standard tools for educational and leisure purposes [1]. While digital devices are increasingly being used in classrooms, optimizing their use to facilitate the best educational outcomes remains a challenge [2]. Given the strong appeal of digital-based learning and assessment, many educational systems have already adopted these technologies [3]. Moreover, there is even some support for encouraging paperless classrooms [4]. Consequently, contemporary student learning and assessment practices are being transformed. Students now employ digital screens in various ways, including studying (e.g., reading digital material, using online educational platforms such as Moodle), connecting with peers, and communicating with teachers. Notably, during the height of the COVID-19 pandemic, all or the majority of schooling was conducted online using digital devices and online communication platforms like Zoom or Microsoft Teams. This has fostered a distinct reevaluation of teaching strategies and methods in education [5]. Thus, there is a growing need to analyze and comprehend digital-based and paper-based learning practices.

One domain that has been substantially impacted by technological advancements and its daily usage is reading. Reading habits have undergone a substantial shift, as students have become more inclined and accustomed to reading on digital devices (i.e., electronic reading) compared to traditional paper reading methods [6]. International large-scale assessments, such as the IEA’s Progress in International Reading Literacy Study (PIRLS), are beginning to administer tests through digital means. For example, the 2016 cycle of PIRLS allowed for the possibility of participating countries using paper-based and computer-based modes (ePIRLS) of assessment (with the subsequent possibility to compare those two modes), while digital formats are encouraged for future use. For the 2021 cycle, ePIRLS is already included in the PIRLS Digital proposal, although a comparison between the two modes is not possible anymore as it was an option in the 2016 cycle. It is crucial to perform a bridge study to be able to compare data from countries which still administer the study on paper with those on computers; this is necessary because of trends analyses as well (to be able to compare reading literacy between different cycles).

As pointed out almost 20 years ago [7], the reading behavior of students is changing, with screen-based reading becoming a popular trend among students who have started using digital screens at an early age (i.e., those growing up in the contemporary digital world that are frequently identified as digital natives). Similarly, recent research [6] concluded that digital-based reading is unavoidable and an integral part of the modern educational landscape. However, going digital may not always be best suited for fostering deep comprehension and learning. Therefore, comprehensively understanding the topic is essential to appreciate the benefits and be aware of the challenges and issues associated with digital-based and paper-based learning practices. Hence, the goal of this paper is to provide a clear understanding of the current state of research and findings regarding digital reading versus paper reading outcomes in students aged between 6 and 18 years, which corresponds to the different populations assessed in international large-scale student assessments. Furthermore, we aim to assess the “impact” of associated factors (gender, socioeconomic status, and urban/rural school location) in explaining the differences between paper and digital reading outcomes. This corresponds to an urgent call to action [8], as there is a definite need for a greater understanding of the individual, social, and environmental factors that might jointly contribute to the competence of students in reading, especially as it pertains to digital reading performance. 

Paper and electronic/digital reading are defined as distinct constructs. According to Walsh [9], paper reading can be operationalized in two ways. First, it includes reading text due to a motivation by a need to find specific information or facts for a specific purpose. Second, it entails the linear reading of a text with concentration and emotional engagement, whereas digital reading encompasses digital literacy, which refers to the combination of text and other multimedia resources available exclusively within an electronic, digital context. Digital literacy does not just pertain to text read on a digital device [10,11,12], but it incorporates multimodality. Moreover, the current state of research on digital reading is not without its challenges [13]; there is a lack of conceptual clarity, with many studies failing to define what digital reading is and what it entails in their research designs.

The aforementioned observations highlight the importance of recognizing the variations and disparities present in the literature when comparing paper reading to digital reading. In the past, digital reading primarily referred to reading text documents, such as PDFs or DOCs, displayed in a digitalized manner on screens. However, the field has recently shifted its focus to encompass reading in an online, digital, and multimodal environment [14]. Nevertheless, a substantial debate persists regarding the effectiveness, similarities, and differences between paper and digital reading, with researchers yet to reach a consensus [15].

As emphasized by Naumann and Saelzer [16], both paper and digital mediums involve several cognitive operations and corresponding component skills, including decoding, syntactic parsing, semantic integration, building textbase, and situational mode. However, certain skills associated with digital reading do not have an equivalent when assessing paper reading [17]. For instance, as digital texts are presented on devices such as computers, tablets, or mobile phones, basic computer skills become a requirement for digital reading [16] which are not required for traditional paper reading. Moreover, digital reading in an online environment necessitates making strategic decisions regarding which texts to read and the order in which to read them [18]. Additionally, digital reading involves a distinct process where text comprehension and navigating digital environments intersect, setting it apart from its paper counterpart. This process, often referred to as “navigation”, involves hypertexts and the combination of multiple interconnected layers or levels of text, requiring nonlinear reading from beginning to end, unlike paper texts that are normally read linearly [16,18]. Consequently, readers are tasked with selecting and integrating different elements of the text during digital reading, we can say that they need to find their own paths of reading, which is normally not present in traditional paper reading. 

Several systematic reviews have already been conducted to compare paper-based reading with digital reading [1,6,15]. Clinton [1] found a negative effect of screens on reading performance compared to reading on paper, particularly in the case of expository texts, while no differences were found for narrative texts. Delgado et al. [6] observed an advantage of paper over digital reading in studies employing both between-participants and within-participants study designs. The findings were moderated by factors such as time frame, text genre, and publication year. Finally, Kong et al. [15] established that reading on paper led to better reading comprehension compared to reading from screens, with no significant differences in terms of reading speed between the two modes. These studies provide some intriguing evidence for the advantages of paper-based reading over digital reading. However, it is important to note that two out of the three studies [1,6] focused on undergraduate and adult samples, while the review by Kong et al. [15] was in fact primarily concentrated on post-secondary students but also included studies with mixed-sample designs (university students, adults). Consequently, based on this, we can say there is a clear need for a systematic review that specifically targets primary and secondary school students (aged 6–18 years), which aligns with the population assessed in international large-scale student assessments (ILSAs).

### 1.1. Problem Statement

As already shown above, reading on electronic devices is becoming widespread and is not something new, but it has become increasingly popular and widespread over recent decades. Electronic sources are often online, and multimodal—containing not only text but image and video content [14]—because they are based in a digital environment. The organization of the reading content, however, leads to different reading strategies taken by the reader, as well as different underlying cognitive processes compared to traditional paper reading. Until the introduction and use of electronic assessments tests of reading abilities [19], it was unknown how big the gaps in reading literacy between electronic and paper reading are. The Progress in International Reading Literacy Study (PIRLS), 2016 [20], was one of the first studies conducting assessments of students with reading tests in both paper and electronic modes. PIRLS 2016 found a large variation in the students’ reading performances between paper and electronic informational reading in countries taking both modes of administration, especially between the two genders [21]. Similarly, it has been reported in the USA that students who take the computer-based version of the standardized exam PARCC (Partnership for Assessment of Readiness for College and Careers) score lower on the test compared to those who take it on paper. This holds true for the assessments of both mathematics and English/language arts. This can be described as the digital divide in regard to reading from digital- and paper-based means (for a comprehensive contemporary overview of the topic refer, to [22,23,24]), which can be seen to contribute to the already-evident achievement gap between different students.

While there have been some attempts to explain the gaps between students based on some student characteristics and behaviors in some countries, see [14], there have not been many thorough analyses. In addition, although there are some sources on the differences in reading comprehension with the different reading modes, there is no comprehensive literature review for the here-studied age group of students (6–18 years). This paper aims to fill these gaps. Therefore, our research question is as follows: What differences have been highlighted in recent literature comparing digital and paper reading among students 6–18 years old focusing on factors of gender, socioeconomic status, and school location (urban and rural)?

### 1.2. Contribution to the Field and Objectives

Although electronic reading itself is not a new phenomenon, the research on differences between paper and electronic reading is. The reason for this is the relatively new implementation of ability tests using electronic reading materials [19]. This systematic review aims to contribute to the field through a comprehensive overview or description of the current state of digital vs. paper reading in recent publications. The paper also has an additional focus on the factors contributing to the gaps in the two reading modes. These additional factors are gender, socioeconomic status, and the school location (rural/urban) where participating students study. The review of the literature covering the differences in electronic reading on these factors also sheds light on the gaps in research on the gaps themselves.

Based on this discussion, the present research has two main objectives: (1) assessing the state of research comparing digital reading and paper reading outcomes in students aged 6–18 years old; (2) evaluating the impact of various factors, namely gender, socioeconomic status, and school location, as variables that contribute to explaining differences between the two modes of reading.

## 2. Methods

This systematic review uses the recommendations of the Preferred Reporting Items for Systematic Review and Meta-Analysis (PRISMA) statement [25]. The review and its protocol were not previously registered.

### 2.1. Search Strategy

A thorough literature search was conducted (August 2022) using the following electronic databases: Web of Science, Scopus, ERIC, and JSTOR. Furthermore, the initial search was expanded through backward citation chaining and an additional search in Google Scholar to include the latest research in order to foster a comprehensive overview of the research field.

The selection of these databases aimed to encompass a broad range of search results. The search terms were formulated based on the purpose of the paper and specific inclusion criteria, incorporating terms such as “digital reading”, “internet reading”, “e-reading”, “digital literacy”, “online reading”, and “digital divide.” Additionally, factors that may influence e-reading versus paper-reading outcomes, such as “SES”, “gender”, “urban”, “rural”, and “socio-demographic” were included. However, for the purpose and scope of this paper, the factors were limited to students’ gender, socioeconomic status (SES), and the urban/rural location of their schools.

Table 1 provides an overview of keywords used in the respective databases. As each database has its own specifics, the search string used is based on the requirements of that specific database while also incorporating terms searched for in a way that matched between the databases.

### 2.2. Eligibility Criteria

Table 2 offers an overview of all criteria that determined whether a study was included in the systematic review.

Studies had to focus on the appropriate age group, which was defined as children/adolescents aged 6–18 years old. This age range was defined for two reasons. First, to include all possible age ranges of students assessed in international large-scale assessments (for example IEA’s PIRLS assessed students at age 10; OECD’s PISA (Programme for International Student Assessment) assessed students at age 15) and secondly to correspond to ISCED (International Standard Classification of Education) levels 1, 2, and 3. Studies that included participants outside of this age range were excluded.

The research focus of included studies had to be on both e-reading and paper reading as this was a prerequisite to provide a comparison between the included studies. This corresponds to the main objective of the present systematic review of assessing e-reading and paper reading outcomes. Studies were excluded if they were focusing only on one construct or if the research focus was not placed on e-reading and paper reading. 

For a study to be included, it had to follow either a quantitative or mixed-methods research design. From a mixed-method study, only results from quantitative analysis were deemed appropriate for inclusion. Studies focusing on qualitative analysis were excluded.

Publication type was another aspect to consider. As the nature of e-reading is changing, with multi-modality being in focus we included peer reviewed studies from 2015 onwards. This corresponds to the 2016 cycle of PIRLS that already included e-reading tasks. Studies conducted before 2015 or that were not peer reviewed were excluded. 

Finally, study language was included as a criterion for inclusion. Only studies in English were considered to be included in the present systematic review. Studies not in the English language were excluded. 

### 2.3. Study Selection Process

**Figure 1:**
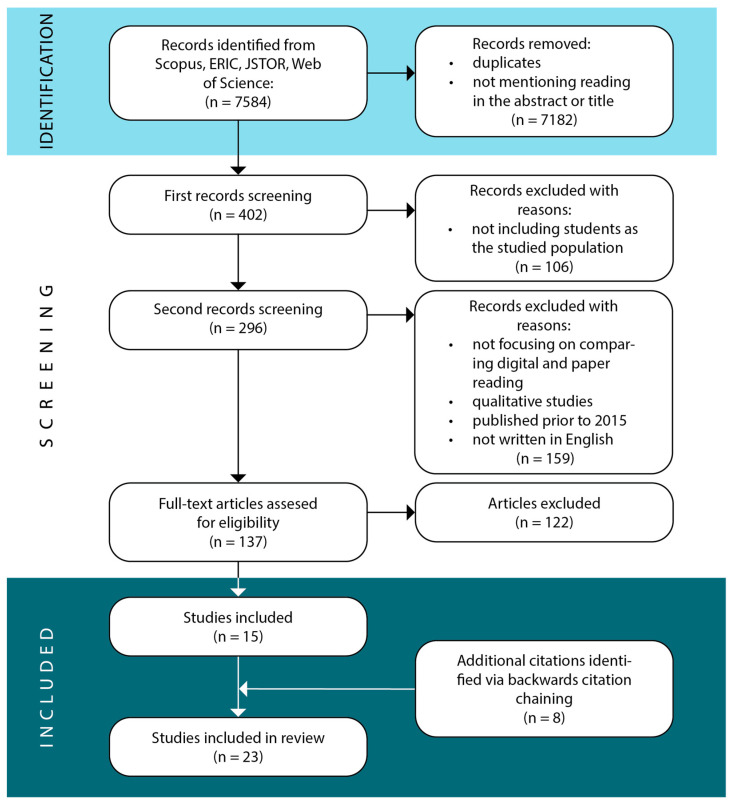
Article search and selection process following PRISMA guidelines.

In total, 23 studies were included in the final sample. Figure 1 depicts the steps of the study selection process and the reasons for exclusion. The figure was modeled according to PRISMA (Preferred Reporting Items for Systematic Reviews and Meta-Analyses) recommendations [25].

The search in all databases followed the same procedure. Three reviewers participated in the study-selection process. As a first step, the literature search was performed in all databases with duplicates being deleted. Secondly, studies were first assessed based on abstract reading only. Reviewers were blind between their respective decisions and discrepancies were resolved by means of discussion. This was carried out to minimize risk of bias between the reviewers. In the third step, full-text reading was conducted and inclusion criteria were assessed. Again, reviewers were blinded to each of their respective decisions to eliminate potential bias in the selection process. All doubtful cases were resolved by means of group discussion. Microsoft Excel was used to record reviewer decisions. A detailed description of the study-selection process follows. 

Initially, the search yielded a total of 6193 results from Scopus, 168 from ERIC, 921 from JSTOR, and 302 from the Web of Science library. In the subsequent step, duplicates were removed, and the focus was directed towards articles that mentioned reading in either the title or abstract, resulting in a narrowed down set of 402 articles. In the third step, articles that did not involve students as the studied population were excluded, further reducing the number of results to 296. In the fourth step, abstracts were read again and assessed in regard to our inclusion criteria that focused on participants, research focus, study type, publication, and language of publication (presented along with exclusion criteria in Table 2). Based on this, 137 articles were selected for full reading. Based on full reading, 15 articles were selected for inclusion in this review. In addition, eight articles were identified through backward citation chaining from recent meta-analyses [1,6] and included studies [26,27]. Three recent studies included were found using an additional search on Google Scholar, based on the aforementioned search terms presented in Table 1 [28,29,30].

### 2.4. Data Extraction

Microsoft Excel was used to extract data from included studies. Data extraction included author details, study title, year of publication, the aim of the study (research questions included), sample characteristics (age, school level, country of origin), assessment of digital reading (describing the means of assessment of digital reading), and main results (description of main findings relevant to the purpose and objectives of the present systematic review). In alignment with the PRISMA statement [25], special consideration was also given on the reviewed factors (SES, gender, and school location); if a study did not measure or analyze these factors, this was recorded in the extraction table. Findings were checked and discussed by the reviewers to ensure agreement. 

### 2.5. Analyses

We used a systematic literature review methodology, which is a comprehensive and rigorous method of reviewing and synthesizing existing research studies on a specific topic or research question [26]. It involves systematically searching, critically appraising, and analyzing a wide range of relevant sources to provide an unbiased, reliable, and evidence-based summary of the existing knowledge on the subject of interest. The steps used in our study to gather and synthesize evidence from multiple sources, designed to provide a reliable and unbiased summary of existing knowledge on the topic of digital versus paper reading among 6–18-year-old students, are described here: (1) defining research questions; (2) selecting databases and other research sources; (3) defining search terms; (4) merging hits from different databases; (5) applying inclusion and exclusion criteria; (6) performing the review; (7) synthesizing results.

In the process of conducting analyses for the systematic review, the key findings of the included studies were summarized. All studies were summarized according to the objectives of the present systematic review (i.e., to gather a comprehensive overview of the state of e-reading vs. paper reading research among 6–18-year-old students, with special focus on factors that help to explain the possible differences between the two modes of reading including gender, SES, and school location).

### 2.6. Literature Search Results

As mentioned, the literature search yielded 23 papers to be included in the present review. Table 3 presents an overview of the included studies. All papers were published between 2015 and 2021. Two papers were published in 2015 [26,27], two in 2016 [28,29], three in 2017 [10,16,30], three in 2018 [14,31,32], two in 2019 [33,34], five in 2020 [35,36,37,38,39], and six in 2021 [8,40,41,42,43,44]. 

To facilitate the present review, several elements were coded from the studies: the aim of the study, participants (sample size, country of origin, and age), assessment of digital reading (i.e., how digital reading was assessed and/or defined in a particular study), and the main results. More detailed results from the studies are described in the subsequent section. 

## 3. Results

The following paragraphs present the main findings from the reviewed studies. The results are categorized into different sections: comparison of e-reading vs. paper reading in terms of reading comprehension, predictors at the individual level and higher level, gender differences, and socioeconomic differences. Lastly, some additional findings concerning the e-reading vs. paper-reading literature are described. None of the studies focused on school location factors, i.e., urban vs. rural school setting (this is why no results are described or discussed taking this into account).

### 3.1. Reading Comprehension

Most of the included research focused on the aspect of reading comprehension in different reading modes (digital vs. paper). In some experimental studies, there were no differences in reading comprehension found between the two reading modes [27,29,36,41,42]. A secondary analysis of 2016 ePIRLS and PIRLS data from South Africa also revealed no significant differences in mean achievements on paper compared to digital reading [33]. In addition, Porion et al. [29] similarly found no effect of reading mode on memorization. However, if digital texts were presented within an online program, which provided vocabulary explanations, students scored higher on vocabulary questions compared to paper or pdf type texts. Although vocabulary assistance was also available in the latter formats, it remained underutilized, leading to a significant difference in students’ performance [42].

On the other hand, some experimental research also showed that students’ reading comprehension is better on paper compared to digital texts, supporting the hypothesis of screen inferiority [26,32,38,39]. The effect sizes in these studies are small–medium (from *d* = 0.15 to *d* = 0.44). This is consistent with the findings of the secondary analysis performed using German PISA 2012 data, which showed that students exhibit a lower proficiency in digital compared to paper reading [16]. Additionally, Goodwin et al. [37] showed a small advantage of paper reading for comprehension when students read longer texts.

Lenhard et al. [30] studied the effect of reading mode on different levels of performance (at word, sentence, and text levels) in the same reading comprehension test. They found that, although students completed the test faster in the digital condition, they were less accurate. Although results showed that students did perform better in the digital test compared to paper when examining the word level results, no main effect of the mode of reading was found on the sentence and text levels.

#### 3.1.1. Individual Level Predictors of Reading Comprehension

Research also focused on different predictors of digital and paper reading comprehension. Cho et al. [8] reported that paper reading comprehension is one of the best predictors of digital reading comprehension, which means that students who read better on paper also read better digitally. This was also confirmed by Naumann and Saelzer [16], who found a high positive correlation (*r* = 0.80) between proficiency in digital and paper reading. Additionally, in a Spanish experimental study, Salmerón et al. [43] showed that, although the main effect of reading mode was not significant, the interaction with students’ prior comprehension skills and time pressure was significant. Students with high reading comprehension skills demonstrate comparable levels of text comprehension when reading on tablets as they do when reading in print mode, even under time pressure, but students with low comprehension skills had difficulties comprehending digitally presented texts on tablets under time pressure. In contrast, a study by Støle et al. [39] showed that highly skilled readers lose more information compared to low-skilled readers when taking the test digitally compared to paper.

Duncan et al. [28] examined the predictors of general reading comprehension by looking into the reading habits of participants. Their research revealed that although the reading habits of students showed a tendency for more frequent exposure to digital than paper texts, more positive associations with reading comprehension and related cognitive constructs (fluency, word identification) were found for paper compared to digital texts. Thus, reading comprehension and related cognitive constructs were stronger predictors for paper reading scores. In contrast, a study of Dominican Republic secondary school students revealed no association between digital academic reading habits and reading competencies [10].

Goodwin et al. [37] also looked into the strategies used in digital and paper reading. Their results indicated that the number of highlights students made on paper negatively predicted reading comprehension, while the opposite pattern emerged for digital texts. Additionally, looking back (i.e., retrospectively searching the text for an answer) at paper text positively predicted comprehension, which was not observed for digital texts.

The effect of different motivational constructs on reading comprehension was also explored in different studies. For example, international large-scale assessments like (e)PIRLS include such measures in their testing. In examining their validity, Cho et al. [8] found that reading self-concept was a significant predictor of reading comprehension in both reading modes, although this effect was found to be stronger for comprehension in paper-based format. Liman Kaban and Karadeniz [42] investigated a related concept, namely perceived self-efficacy during the performance of the reading task. They found that students perceived themselves as more self-efficient in the digital compared to the paper reading mode. It was also hypothesized that readers’ attitudes can have an important effect on comprehension. In the secondary analysis of PIRLS data, attitudes toward reading were not a significant predictor of reading comprehension in any of the reading modes [8]. On the other hand, Jang and Ryoo [34] examined the attitudes specifically toward the reading mode of the text. They found a strong positive relationship between attitudes toward paper mode and comprehension but did not find this relationship for the digital mode. However, in an Israeli study [38], no effect of prior preference for reading mode on comprehension was observed.

#### 3.1.2. Higher-Level Predictors of Reading Comprehension 

On the family level, the number of books at home is a strong predictor of paper reading comprehension and is significant in predicting digital reading comprehension [8,14]. In addition, parental enjoyment of reading, early-life home literacy activities, and parental education are associated to a similar degree with results on digital ePIRLS and the paper-based PIRLS achievements in reading literacy [14].

On the systemic level, the usage of ICT resources in schools was one of the strongest predictors of paper-based and even stronger predictor of digital reading achievement in the South African ePIRLS and PIRLS 2016 sample [33]. However, in Ireland, the frequency of computer usage in school was a significant positive predictor of reading literacy on the digital ePIRLS 2016, but not on the paper-based PIRLS 2016 reading literacy achievements [14]. This research also showed that less frequent general computer use is associated with higher reading literacy achievement in both reading modes.

### 3.2. Gender Differences 

Studies on gender differences in paper vs. digital reading yield mixed results on different aspects of reading. The majority of research comparing reading comprehension between male and female students suggests that there is no difference between the two groups. Cho et al. [8] analyzed the data from the (e)PIRLS study 2016 from the USA and found that gender did not predict digital or paper reading comprehension. The secondary data analysis of ePIRLS and PIRLS 2016 in South Africa yielded similar results [33]. An experimental US study by Goodwin et al. [37], who compared reading comprehension and the number of highlights and annotations on paper vs. digital format, also showed no effect of gender or its interaction with SES on reading comprehension in both reading modes. Similarly, an experimental study from Støle et al. [39], aiming to provide a reliable digital reading test, showed no gender differences in different modes of assessment, since both genders achieved higher scores in the paper compared to the digital condition. There were also no significant differences found in an Israeli [32] and a South African study [27].

From a different point of view, there are some studies that reported significant gender differences in reading comprehension. Naumann and Saelzer’s [16] secondary analysis of the German PISA 2012 data showed that female students outperformed male students in digital reading; they did not check for gender differences in paper reading. A Norwegian study [36] showed that females outperformed male students in the digital mode, but there were no gender differences found in the paper reading mode. This was confirmed by a significant interaction effect between reading mode and gender, which showed that differences in test scores between male and female students were larger (in favor of female students) when the test was administered in a digital compared to a printed format. Similar results were obtained in a Swedish study [26], where female students performed better on paper (*d* = 0.07) and even better digitally (*d* = 0.22) compared to male students. However, the effect sizes reported in the study have to be considered as small. These results suggest that the possible gender gap in reading increases when a digital format is provided to the students. On the other hand, it has also been shown that the highest-performing female students lose the most in terms of comprehension when reading digitally compared to paper [45] showing that gender differences can be complex to understand when detected.

Research also reports some gender differences in specific attitudes toward digital and paper reading. A study by Eijansantos et al. [35] reported that female students showed more positive attitudes toward print text compared to male students; however, no differences in attitudes toward digital reading were detected. Some studies have focused not only on the reading mode of text (digital vs. paper) but also on its content (academic vs. recreational) [34,40]. A study using a person-centered approach by Jang et al. [40] investigated latent profiles based on readers’ attitudes toward both reading modes and showed that gender significantly predicted profile membership probabilities. Female students were more likely to be classified as avid readers with high positive attitudes towards digital and printed academic and printed recreational texts, as well as being willing readers with average attitude levels towards digital/printed academic and recreational texts. In contrast, male students were more likely classified as print-preferred readers or reluctant readers with negative attitudes toward digital and printed academic and printed recreational texts. Similarly, a different variable-centered study focusing on the same attitudes found that female students showed more positive attitudes toward print and digital recreational reading, but no differences in print and digital academic reading were found [40].

### 3.3. Socioeconomic Status Differences 

Studies on socioeconomic status (SES) differences in paper and digital reading are scarce. Most of the evidence for the effect of SES on reading comprehension comes from secondary analysis of international large-scale student assessments. A German secondary analysis of PISA 2012 [16] revealed that SES explains a substantial proportion of variance in digital reading scores (10%), but this variance was even greater in paper reading scores (15%). SES was a significant positive predictor in both cases, showing students with higher SES to have better reading scores. SES differences were also explored in the secondary analysis of the Irish PIRLS and ePIRLS 2016 datasets [14], yielding similar results. In their analysis, SES was again a positive predictor of reading achievement in both modes of assessment. In addition, the same pattern as previously emerged, as the effect size of SES on reading comprehension was larger for the paper mode (*d* = 0.18) compared to the digital mode (*d* = 0.13). However, these effect sizes still have to be considered small. There are also reports of no effect of SES on reading comprehension in different modes of reading from an experimental US study [37], where SES was added as a variable of students’ characteristics, while the authors themselves call for a more nuanced investigation of students’ characteristics’ effects on reading comprehension in different reading modes.

### 3.4. Additional Aspects of Paper vs. Digital Reading

Research also looked into other aspects of reading in different reading modes not directly related to reading comprehension or attitudes, which also have to be taken into account when assessing paper vs. digital reading. Sun et al. [44] studied the effect of the lockdown on reading enjoyment in Singapore. They found that children who enjoyed reading before the lockdown enjoyed reading and read more during lockdown as well. While the opposite pattern was observed for children who did not enjoy reading before lockdown (they read less and they did not enjoy it during lockdown). In addition, there were attempts to investigate different factors affecting reading in different reading modes. Cheng et al. [31] explored visual fatigue related to reading and showed that there is no difference in subjective reports of visual fatigue between digital and paper reading modes and that only reading duration was associated with visual fatigue. One of the hypotheses aiming to explain the difference in performance between paper and digital reading suggests that students get distracted and lack attention when reading digitally. Salmerón et al. [43] found evidence for the opposite by showing that digital reading mode does not effect on-task attention, as reported by participants. 

## 4. Discussion

The comparison between electronic (e-reading) and paper reading has been a topic of interest in educational research, and several studies have investigated various but separate aspects of this debate. The main findings from the reviewed studies can be summarized as follows: reading comprehension differences, gender differences, and SES differences.

For reading comprehension, the results are mixed, with some studies showing no significant differences in reading comprehension between electronic and paper reading modes [27,29,36,41,42]. However, other studies suggest that paper reading may lead to better comprehension compared to digital texts. Dahan Golan et al. [32], Halamish and Elbaz [38], Rasmusson [26], and Støle et al. [39] found evidence supporting the “screen inferiority” hypothesis. Additionally, providing vocabulary explanations within an online program during e-reading can enhance vocabulary-related performance [42]. One could assume that there are differences in supporting the superiority of some modes of reading due to non-representative sample sizes that, for some of the aforementioned studies, due to very small sample sizes, are evident. But it seems that this “reading comprehension mode effect divide” is not even unique when we take into account proven representative samples. As seen from comparing results from ePIRLS vs. PIRLS 2016 cycle (comparing informational reading of grade 4 students; app. 10 years old students) in seven countries (Denmark, United States, Singapore, United Arab Emirates, Norway [grade 5], Israel, and Sweden), a statistically significant difference favors e-reading; in two (Ireland, Canada), there is no statistically significantly difference; and in the remaining five countries (Portugal, Georgia, Italy, Slovenia, and Chinese Taipei), paper reading resulted in higher scores for informational reading compared to e-reading [21]. It becomes clear that there is not a “one type fits all” explanation, as there are differing results even on representative samples of students. 

This is why it is essential to try to identify at least some predictors of reading comprehension. At the individual level, predictors of paper vs. e-reading comprehension include students who perform better in paper reading also tend to perform better in digital reading [8,16]. Students’ prior comprehension skills and time pressure can interact to affect comprehension in digital reading [43]. Reading habits, strategies used during reading, and motivational constructs can also “impact” reading comprehension in different reading modes [37,39,41,43]. At the higher level, predictors of reading comprehension include family-level factors, such as the number of books at home and parental enjoyment of reading, which are associated with both paper and digital reading comprehension [8,14]. The usage of ICT resources in schools is a strong predictor of digital reading achievement [33]. Additional evidence that “one type does not fit” always can be found in the ePIRLS 2016 cycle, as the ePIRLS results comparing two comprehension processes show that Singapore, Chinese Taipei, Portugal, Georgia, and the United Arab Emirates had a relative advantage in retrieving and straightforward inferencing. Only the United States and Canada had a relative advantage in interpreting, integrating, and evaluating. The remaining countries had essentially no difference between the two processes [21]. Other differences can be also attributed to individual- and family-level differences; however, as is already clear, some differences—identified in different countries participating in ILSAs—can be located in curriculum contexts too. 

In terms of gender differences, studies examining gender differences in reading comprehension in different modes have yielded mixed results as well. Some studies report no significant gender differences, e.g., for ePIRLS 2016 in the United States [8] and for the same cycle in South Africa [33]. However, there were also other countries participating in ePIRLS for which our literature review did not find any articles due to our search terms, but for which results are in some cases also different. Both females and males had higher achievement on ePIRLS than PIRLS informational reading in Denmark, Israel, Norway, Singapore, United Arab Emirates, and the United States, and showed higher achievement in PIRLS informational reading in Chinese Taipei, Georgia, Italy, Portugal, and Slovenia [21]. The results by gender were different in Ireland, where the national difference was not significant but females had an advantage in ePIRLS, and in Sweden where the national results showed a difference favoring ePIRLS that was only significant for males [21]. Some others show that the gender gap in reading comprehension may increase in digital reading [16,26,36,39]. Attitudes toward different reading modes may also vary based on gender [34,35,40]. 

Socioeconomic status and other additional differences: SES seems to be positively associated with reading comprehension in both paper and digital modes, although the effect size may be larger for paper reading [8,14,16]. Other factors, such as reading enjoyment, visual fatigue, and on-task attention, were also considered in the studies. Lockdown measures during the COVID-19 pandemic affected reading habits, with children who already enjoyed reading before the lockdown continuing to do so during the lockdown [44]. Visual fatigue did not significantly differ between digital and paper reading [31]. Contrary to the distraction hypothesis, digital reading did not negatively effect on-task attention [43].

Overall, the comparison between electronic and paper reading modes is complex and multifaceted, with various factors influencing reading comprehension and attitudes toward different reading formats. The findings suggest that individual differences, such as reading habits and strategies, as well as family, play significant roles in shaping reading performance in both electronic and paper formats. In our paper, we did not find results on school-level factors (besides usage of ICT in schools) when discussing possible differences between digital and paper reading; our search terms included school location (rural vs. urban), but no results for this factor were found in the databases that we used. However, there are other studies that found the association between SES and school location in reading gaps from a previous PIRLS study (the 2006 cycle to be precise), where schools in urban areas outperformed schools in rural areas, and the gap between these types of school is fully accounted for by the SES composition of the student intake [45]. However, no differences between digital and paper reading at that time were investigable. It is certain that there is no way back when we think about the importance of digital reading inside and outside of schools. This is why, in the future, special attention should be given to digital pedagogy when we think about digital reading in schools. Online reading involves being able to use reading comprehension skills and strategies in contexts that are very different from those encountered in reading traditional printed materials [46]—strategies that are new to digital immigrants (i.e., parents and other relatives of today’s school population, not to mention a large number of teachers also).

## 5. Conclusions

In conclusion, the literature on electronic vs. paper reading provides diverse findings, indicating that the “impact” of reading mode on comprehension and related factors is nuanced and context-dependent. The evidence does not unequivocally support the superiority of one mode over the other. Instead, the relationship between reading modes and reading performance is influenced by individual-, family-, and school-level factors. Future research in this area should consider a more nuanced investigation of the effects of different variables and explore potential interactions between various factors to gain a comprehensive understanding of electronic vs. paper reading. Additionally, as technology and reading habits continue to evolve, ongoing research will be necessary to keep abreast of the dynamic relationship between reading modes and reading comprehension.

Comparing digital and paper reading has inherent limitations due to the complexity of individual preferences, evolving technologies, and varying factors that can influence reading experiences. To provide a comprehensive analysis of this topic, it is essential to acknowledge and address these limitations carefully. However, our paper focuses on some limited factors, such as reading comprehension, gender, and SES differences (including other family factors such as parents’ attitudes towards reading), and the lack of focus on school-related factors (besides the usage of ICT resources) in the literature. Indeed, none of the studies reviewed had a primary focus on school location factors, i.e., the urban vs. rural setting of the participating schools. Besides the language limitation (as only English publications in selected databases were included), another limitation of our study is the lack of focus on health issues when comparing those two modes of reading. Some studies have raised concerns about potential adverse health effects related to excessive digital reading, such as disrupted sleep patterns due to blue light exposure before bedtime. This aspect is worth considering in any comparison to understand the potential impact on readers’ overall well-being. There are some other limitations which are highly relevant to our study, namely that future research on differences between digital and paper reading can and should address—in addition to its impact on memory and retention—access and equity of access to paper sources and digital devices (e.g., economic disparities and geographical limitations), screen fatigue, eye strain, etc. The landscape of digital reading is continually evolving with new hardware and software being developed regularly. This rapid pace of technological advancement means that a comparison made at one point in time may become outdated relatively quickly. At the same time, it also means that we need to devote more and more time to this area in the future, both in and out of school. This is why more in-depth analysis with more complex modeling of the association of different school and out-of-school factors is needed and will be continuously required in the future. However, the factors we were interested in in our systematic literature review (SES, gender, and school location) have not been studied much in the studies reviewed, so there is room for further empirical analysis here too.

## Figures and Tables

**Table 1:** Search strings used in databases.

Database	Search String Used
Web of Science	TS = ((“digital reading” OR “internet reading” OR “e-reading” OR “digital literacy” OR “online reading”) AND (ses OR gender OR urban OR rural OR “digital divide” OR socio-demographic)) AND PY = (2015–2022)
Scopus	((“digital reading” OR “internet reading” OR “e-reading” OR “digital literacy” OR “online reading”) AND (PUBYEAR > 2015) AND (PUBYEAR < 2022) AND (“ses” OR gender OR urban OR rural OR “digital divide” OR “socio-demographic”)) AND (LIMIT-TO (DOCTYPE, “ar”) OR LIMIT-TO (DOCTYPE, “ch”) OR LIMIT-TO (DOCTYPE, “re”)) AND (LIMIT-TO (LANGUAGE, “English”))
ERIC	((“digital reading” OR “internet reading” OR “e-reading” OR “digital literacy” OR “online reading”) AND (“ses” OR gender OR urban OR rural OR “digital divide” OR socio-demographic)) AND (pubyear: 2016 OR pubyear: 2017 OR pubyear: 2018 OR pubyear: 2019 OR pubyear: 2020 OR pubyear: 2021) OR pubyear: 2022)
JSTOR	((“digital reading” OR “internet reading” OR “e-reading” OR “digital literacy” OR “online reading”) AND (“ses” OR gender OR urban OR rural OR “digital divide” OR socio-demographic))

**Table 2:** Inclusion and exclusion criteria for systematic literature search.

	Inclusion Criteria	Exclusion Criteria
Participants	Children/adolescents aged between 6 and 18 years	Not falling into the age range
Research focus	E-reading/paper reading	Not focusing on comparing e-reading/paper reading
Study type	Quantitative or mixed-methods studies	Qualitative studies
Publication	Peer-reviewed; published between 2015 and 2022	Published prior to 2015
Language	English	Not English

**Table 3:** Overview of studies in the systematic review along with main characteristics.

Authors	Aim	Participants	Assessment of Digital Reading	Main Results
Amiama-Espaillat and Mayor-Ruiz, 2017 [10]	Exploring digital reading habits of Dominican Republic teenagers and their connection to reading literacy.	382 Dominican Republic students aged 13–18 (225 female)	Self-reported digital reading frequency scale	No association between digital academic reading habits and reading competencies.
Cheng et al., 2018 [31]	Exploring visual fatigue in reading e-books vs. printed books	24 Taiwanese students aged 11–12 (12 female)	Digital e-books	Positive association between reading duration and visual fatigue, but no association between reading mode and visual fatigue.
Cho et al., 2021 [8]	Secondary analysis of ePIRLS and PIRLS data from USA, exploring the validity of motivational constructs and predictors of reading comprehension (motivational, cognitive, and environmental).	4090 US students aged 9–10	ePIRLS reading comprehension test (assessment of online reading and acquired knowledge implementation)	Paper reading comprehension is one of the largest predictors of digital reading comprehension, while gender was not a significant predictor of any.
Combrinck and Mtsatse, 2019 [33]	Secondary analysis of ePIRLS and PIRLS data from South Africa. Exploring predictors of reading comprehension for both assessments.	12,810 South African students (PIRLS 2016), and 15,744 students (PIRLS 2011) aged 9–10, and 277 students (ePIRLS 2016) aged 10–11 (120 female)	ePIRLS reading comprehension test (assessment of online reading and acquired knowledge implementation)	No difference in paper vs. digital reading mode achievements and gender was not a predictor of any outcomes.
Dahan Golan et al., 2018 [32]	Experimentally exploring reading comprehension, preferences, and self-evaluations in paper vs. digital reading modes.	82 Israeli students aged 10–13 (64 female)	Six texts and a comprehension test administered digitally on a computer screen	Students showed better reading comprehension on paper than digitally.
Duncan et al., 2016 [28]	Investigation of cognitive, psychological, and ecological predictors of reading comprehension for fictitious and non-fictitious texts.	312 UK students aged 7–11 (172 female)	Digital print exposure (i.e., ecological predictor) was collected using a diary method	More positive associations with reading comprehension were found for paper compared to digital texts.
Eijansantos et al., 2020 [35]	Correlational investigation of attitudes toward digital and paper reading with a focus on gender differences.	562 Philippine students aged 17–18 (319 female)	Attitudes towards digital and paper reading	Female students showed more positive attitudes toward print text compared to male students. No gender differences in attitudes toward digital reading were detected.
Engdal Jensen, 2020 [36]	Exploring the effects of administration mode (digital vs. test) on reading comprehension test results with a focus on gender differences.	973 Norwegian students aged 13–14 (473 female)	Two reading comprehension tests, administered digitally on a computer screen	No main effect of reading mode on comprehension; however, female students outperformed male students in the digital condition.
Gilleece and Eivers, 2018 [14]	Secondary analysis of ePIRLS and PIRLS 2016 data from Ireland. Exploring the predictors of paper PIRLS and digital ePIRLS achievements.	2473 Irish students aged 9–10	ePIRLS reading comprehension test (assessment of online reading and acquired knowledge implementation)	SES is a positive predictor of digital ePIRLS and paper PIRLS achievements. Performance on ePIRLS and PIRLS has been found to be associated with home background and home climate variables in similar ways.
Goodwin et al., 2020 [37]	Experimentally investigating differences in annotating and highlighting digital and paper texts, reading comprehension in both reading modes, and their relation.	371 US students aged 10–13 (201 female)	A text and a comprehension test administered digitally on a laptop screen	Positive association between paper highlights and reading comprehension and a negative association between digital highlights and comprehension. Better reading comprehension for longer text in paper mode compared to digital mode. No effect of SES on reading comprehension in any of the reading modes.
Halamish and Elbaz, 2020 [38]	Experimentally exploring reading comprehension and metacognition in paper vs. digital reading modes and its relation to reading mode preferences, digital habits, and reading skills.	38 Israeli students aged 10–11 (22 female)	Four texts and a comprehension test administered digitally on a computer screen	Students showed better reading comprehension on paper than digitally. Their metacognitive judgments did not differ between both reading modes.
Jang and Ryoo, 2019 [34]	Investigating the relationship between reading attitudes (digital vs. print) and gender, achievement, and grade.	586 South Korean students aged 12–15 (272 female)	Attitudes towards digital and paper reading	Positive association between attitudes toward paper reading mode and comprehension, but not for digital mode. Female students have more positive attitudes toward print and digital recreational reading activities.
Jang et al., 2021 [40]	Investigating adolescents’ attitudinal profiles based on attitudes toward print and digital reading.	5080 US students aged 11–13 (2712 female)	Attitudes towards digital and paper reading	Identified four different reading profiles based on attitudes where gender played a significant role in predicting profile membership probabilities.
Lenhard et al., 2017 [30]	Exploring the effects of administration mode (digital vs. test) on reading comprehension test results.	5073 German students aged 7–12 (1365 female)	ELFE reading comprehension test administered digitally on a computer screen	Higher raw scores in digital assessment, although students were less accurate in the digital condition.
Lim et al., 2021 [41]	Experimentally exploring how reading mode of the text and different interactive digital features affect reading comprehension.	30 South Korean students aged 12–15 (16 female)	Six interactive e-books administered digitally	No effect of reading mode on comprehension.
Liman Kaban and Karadeniz, 2021 [42]	Exploring motivational and comprehension differences in reading digital vs. paper texts.	96 Turkish students aged 11–12	Three digital conditions: gamified e-book (students listened, read, took quizzes in an online program); personalized e-book (read, took quizzes in an online program); pdf books (read, took quizzes digitally)	No effect of reading mode on comprehension and higher motivation in digital conditions. In conditions of reading e-books, which provided help (i.e., vocabulary), students showed better comprehension.
Naumann and Saelzer, 2017 [16]	Secondary analysis of PISA 2012 computer-based assessment data from Germany. Comparison of digital and paper reading proficiency on PISA and the relationship between digital reading proficiency and students’ background variables.	5001 German students aged 15 (2462 female)	PISA 2012 computer-based assessment	Lower proficiency in digital compared to paper reading mode. The correlation between both is positive (*r* = 0.80). SES was a positive predictor of reading comprehension in both reading modes.
Porion et al., 2016 [29]	Comparing digital and paper reading comprehension and a set of processes that appear during reading.	72 French students aged 13–15 (40 female)	A text administered digitally on a computer screen and a comprehension test administered on paper	No effect of reading mode on reading comprehension or memorization.
Rasmusson, 2015 [26]	Exploring the effects of administration mode (digital vs. paper) on reading comprehension test results with a focus on gender differences and text format influence.	117 Swedish students aged 14–15 (64 female)	Reading comprehension test (included three texts) administered digitally on a computer screen	Students showed better reading comprehension on paper than digitally. Female students performed better than male students in both conditions; however, the gender gap was larger in the digital mode.
Sackstein et al., 2015 [27]	Quasi-experimentally exploring reading comprehension in paper vs. digital reading modes.	54 South African students aged 15–16 (25 female)	Two texts administered digitally on a tablet	No effect of reading mode on comprehension and no significant gender differences were found.
Salmerón et al., 2021 [43]	Experimentally exploring reading comprehension and attention in paper vs. digital reading modes under time pressure.	182 Spanish students aged 10–13 (78 female)	Two texts administered digitally on a tablet	Students with high reading comprehension skills demonstrate comparable levels of text comprehension when reading on tablets as they do when reading print, even when faced with time constraints. Conversely, students with lower comprehension skills encounter challenges in understanding texts on tablets when time is limited.
Støle et al., 2020 [39]	Experimentally providing a reliable digital reading test and exploring differences in test results in digital and paper reading modes.	1139 Norwegian students aged 10	Two reading comprehension tests (each included five texts) administered digitally on a computer screen	Students showed better reading comprehension on paper than digitally. Female students with high comprehension skills experience the greatest decline in comprehension when reading digitally as opposed to reading from paper.
Sun et al., 2021 [44]	Exploring the effect of lockdown on reading amount, enjoyment, and resources.	2012 Singaporean students 10–11 (973 female)	Self-report measures of digital reading enjoyment, habits, resources	During the COVID-19 lockdown, children who previously enjoyed reading continued to enjoy the activity and increased their reading frequency. Conversely, children who lacked reading enjoyment prior to the lockdown read less and did not develop enjoyment for it during that period.

## Data Availability

No new data were created. Results are based on existing articles on the topic.
